# Modulation of humoral immune response to oral BCG vaccination by *Mycobacterium bovis BCG *Moreau Rio de Janeiro (RDJ) in healthy adults

**DOI:** 10.1186/1476-8518-4-4

**Published:** 2006-09-06

**Authors:** Renata Monteiro-Maia, Maria B Ortigão-de-Sampaio, Rosa T Pinho, Luiz RR Castello-Branco

**Affiliations:** 1 Centro de Pesquisas Arlindo de Assis, Fundação Ataulpho de Paiva, Avenida Almirante Barroso, 54, 15°. Andar, Rio de Janeiro, Brazil; 2 Cellular & Molecular Medicine (Centre for Infection), St. George's, Cranmer Terrace, London SW17 0RE, UK; 3 Laboratório de Imunologia Clínica, Departamento de Imunologia, Instituto Oswaldo Cruz, Fundação Oswaldo Cruz, Avenida Brasil, 4365, Manguinhos, Rio de Janeiro, Brazil

## Abstract

**Background:**

Oral administration of BCG was the route initially used by Calmette and Guérin, but was replaced by intradermal administration in virtually all countries after the Lubeck accident. However, Brazil continued to administer oral BCG Moreau RDJ, which was maintained until the mid-1970s when it was substituted by the intradermal route. Although BCG vaccination has been used in humans since 1921, little is known of the induced immune response. The aim of this study was to analyse immunological responses after oral vaccination with *M. bovis BCG Moreau RDJ*.

**Methods:**

This study in healthy volunteers has measured cellular and humoral aspects of the immunological response to oral *M. bovis BCG Moreau RDJ *in Rio de Janeiro, Brazil. T-cell trafficking and Th_1 _and Th_2 _cytokine responses are described, as well as isotype-specific antibody production using novel techniques.

**Results:**

Oral immunisation has no adverse effects. We have shown that there are cellular and humoral immunological responses after oral immunisation. Oral revaccination does not induce a positive skin test in responsive individuals and multiple booster orally was able to induce modulation in humoral immunological responses (switch from IgG to IgA) in previously immunised subjects and incapable of inducing tolerance. In contrast, the cellular immune response does not differ between vaccinated individuals with positive and negative skin test reactions.

**Conclusion:**

All subjects, including those who did not respond to the skin test at study commencement, were capable of mounting humoral and cellular immune response to the antigens tested.

## Background

BCG vaccination was developed by attenuation *in vitro *over 13 years from a virulent sample of *Mycobacterium bovis *by Albert Calmette and Camille Guérin at the Pasteur Institute, Lille. The attenuated strain named BCG (Bacillus of Calmette-Guérin) is now known as *Mycobacterium bovis BCG*. BCG was given to humans for the first time in 1921, since when it has become the most used vaccine in the world [6]. It has been given to 3 billion people with low incidence of serious adverse events [18]; more than 100 million people currently receive the vaccine in order to prevent tuberculosis [23]. More than 90% of global production is made of the Russian BCG-I, Tokyo 172-1, Danish 1331, Moreau RDJ and Pasteur 1173-P2 sub strains [19].

Despite use of the vaccine for more than 80 years, several controversies remain concerning efficacy, with studies reporting protection rates varying between 0 and 80% [12,13,4,11].

Oral administration was the route initially used by Calmette and Guérin, but was replaced by intradermal administration in virtually all countries after the Lubeck accident, in which 67 of 249 babies given the vaccine died due to contamination of the BCG with virulent *Mycobacterium tuberculosis *[2]. However, Arlindo de Assis continued to administer oral BCG, which was maintained in Brazil until the mid-1970s when it was substituted by the intradermal route. Even after this change in route, the Fundação Ataulpho de Paiva continued to produce the oral vaccine [7].

Studies by Assis and Carvalho [3] showed that none of 167 children developed a response to skin testing one week after oral immunisation; skin-test positivity only appeared from 6 weeks after oral immunisation with BCG Moreau.

It is known that induction of the mucosal immune response is vital for protection against infectious agents whose route of entry is via the mucosa, as is the case for tuberculosis. Oral administration was shown capable of inducing a more substantial mucosal and systemic immune response compared to the intradermal route [16].

Brown et al (2003) [9] showed that BCG could induce mycobacteria-specific antibodies and Williams et al (2004) [26] confirmed that oral vaccination with BCG induced significant increases in IgA isotype anti-LAM antibodies that had important functions in systemic responses as well as offering mucosal protection.

Host resistance to mycobacterial invasion is associated principally with generation of cellular immune responses [15].

CD4^+ ^T cells become activated after presentation of mycobacterial antigens in association with class II MHC molecules, producing cytokines, principally IFN-γ, the principal activator of macrophages [25] that acts in conjunction with TNF-α to recruit macrophages, augmenting the effectiveness of host immune responses [22].

CD8^+ ^T cells are also capable of secreting cytokines including IFN-γ, TNF-α, IL-2 and IL-4 and are important in controlling the equilibrium between Th1 and Th2 responses [25]. Deficiency of these cells provokes poorly organised cellular infiltrates suggesting their importance in the formation of protective granulomas [1]. In addition, these cells appear to have an important role in protection against reactivation of latent infection [21].

Once mycobacteria become intracellular pathogens, serum components cannot gain access and lose their protective function [25]. B-cells have been described as an important source of chemokines involved in granuloma development and consequently inhibit mycobacterial dissemination, resulting in recruitment of appropriate cells to the locality for the first few weeks after infection [8].

The concept of a common mucosal-associated system regulating and coordinating immune responses at mucosal surfaces has been an important advance in our understanding of protection against mucosal pathogens. This system is based on primed T and B lymphocytes that migrate from the site of antigen presentation via lymphatics and blood to selectively "home" to lymphoid tissue at distant sites in gastrointestinal, respiratory, genitourinary and other mucosa-associated regions [17].

In this context, the objective of this study was to analyse humoral and cellular immune responses to mycobacterial antigens and correlate them to the PPD skin tests in healthy adult volunteers in Rio de Janeiro (Brazil) after oral vaccination with *M. bovis BCG Moreau RDJ*.

## Methods

### Volunteers and skin testing

Healthy subjects aged 18–50 years were recruited who gave no history of pulmonary illness, tuberculosis or Hansen's disease, and in whom clinical examination and chest X-ray were within normal limits. For PPD skin testing, Multi-test (Mérieux^®^) was applied to the right arm and read after 48 hours. Volunteers were retested for skin reactivity at the end of the study (6 months after oral immunisation). Written informed consent was obtained from participants before study enrollment.

### Antigens

Antigens used were *M. bovis BCG *vaccine Moreau RDJ, secreted proteins from *M. bovis BCG *Moreau RDJ culture and PPD Rt48 (*Statens Serum Institute*).

### Vaccination

Over a 90 minute period of fasting subjects received 100 ml 2% sodium bicarbonate to neutralise gastric acid and after a gap of 15 minutes were orally immunised with 5 ml *M. bovis BCG *Moreau RDJ vaccine at a concentration of 20 mg/ml (made by Fundação Ataulpho de Paiva).

### Blood collection and collection of peripheral blood mononuclear cells (PBMC)

Blood samples were taken before immunisation (counted on a negative scale), on the day of immunisation (day zero) and on days 3, 5, 7, 10, 12, 14 and 19 after oral immunisation with *M. bovis BCG Moreau RDJ*. PBMC were separated from heparinised blood by a density gradient and lymphocytes were washed and counted prior to use in experiments.

### ELISpot

Between 5 × 10^5 ^and 1 × 10^6 ^PBMC were transferred to 25-well plates pre-coated with 20 μg/ml oral *M. bovis BCG Moreau RDJ *vaccine and incubated for 2 1/2 hours at 37°C in 5% CO_2_. Anti-IgA (2 μg/ml), anti-IgG (2 μg/ml) and anti-IgM (4 μg/ml) anti-human antibodies were added and incubated for 2 hours. Goat anti-human antibody conjugated with alkaline phosphatase (2 μg/ml) was incubated for 2 hours prior to development using BCIP. Wells were read under an inverted microscope after 3 hours of development.

### Cellular lymphoproliferation

U-shaped 96-well plates were coated with secreted BCG proteins or PPD Rt48 (both 10 μg/ml); mitogen used was concanavalin A (2.5 μg/ml). Mean ^3^H incorporation (cpm) was measured in triplicate by scintillation counting in a Beta counter for 1 minute.

### Cytokine measurement

Supernatants of cellular lymphoproliferation assays were frozen at -70°C for subsequent measurement of cytokines IL-4 and IFN-γ using highly sensitive kits RPN-2783 and RPN-2787 (Amersham^® ^LIFE SCIENCE) respectively according to manufacturer's instructions.

## Results

### Skin test responses and correlation with vaccine history

**Figure 1:**
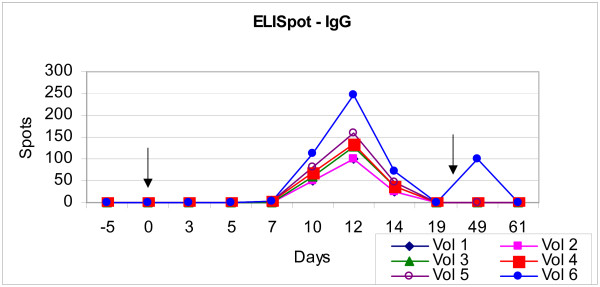
**ELISpot IgG**. Although differences in the number of *spots*, all subjects displayed a peak humoral immune response, observed by the detection of IgG. Arrow indicates time of immunisation and booster.

**Figure 2:**
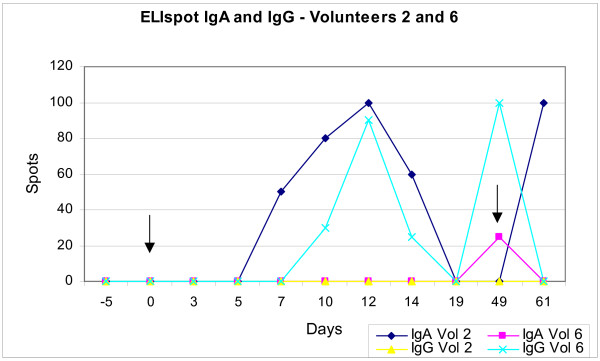
**ELISpot IgA and IgG – Volunteers 2 and 6**. Subjects 2 and 6, who received more than one dose of oral vaccine and had been immunised in infancy via the oral and intradermal routes respectively, displayed an altered humoral immune response with antibodies of isotype IgA. Arrows indicate time of immunisation and booster.

Of 100 individuals assessed for PPD skin reactivity, 77 (77%) were positive and 23 (23%) negative (figure 1). Of the 23 PPD negative individuals, 19 had been immunised orally and 3 intradermally in infancy and 1 had not been immunised (figure 2). Only 6 subjects consented to the immune response kinetic study (Table 1) and just two of them agreed to have an oral booster vaccination 42 days after primary immunisation. Of these 6 subjects, 3 gave a history of contact with tuberculosis (subjects 2, 5 and 6). All subjects were tested for PPD 6 months after oral immunisation and the results were similar to the first tests. Subjects who responded positively to skin testing were evaluated clinically and none showed symptoms or signs of infection.

**Table 1:** Clinical characteristics of the volunteers.

Volunteer	Age	Gender	PPD result	Chest X-Ray	TB exposure	First Immunisation
1	50	F	-	Normal	NO	Oral
2	40	M	-	Normal	YES	Oral
3	33	F	-	Normal	NO	Oral
4	23	F	-	Normal	NO	I.D.
5	25	F	+	Normal	YES	I.D.
6	35	F	+	Normal	YES	I.D.

### Humoral response (ELISpot)

Using the ELISpot technique it was possible to observe the quantity and isotype of antibody production by plasmablasts in response to oral immunisation with *M. bovis BCG Moreau RDJ*. Results were expressed in terms of the number of *spots *formed per 10^6 ^PBMC. Despite differences in the number of *spots*, all subjects displayed a peak humoral immune response, observed by the detection of IgG antibodies, between day 10 and 14 after oral immunisation (figure 1). Subjects 2 and 6, who received more than one dose of oral vaccine and had been immunised in infancy via the oral and intradermal routes respectively, displayed an altered humoral immune response with antibodies of isotype IgA; subject 2 displayed earlier and greater expression on day 12 after immunisation and 12 days after boosting with oral vaccine (day 49) appeared a new peak of IgA expression. Subject 6 (primary Id vaccination at birth) presented IgA immune response after oral immunisation and kept expressive levels of IgG after oral immunisation. In contrast volunteer 2 (primary oral vaccination at birth) kept the previous expression of IgA and presented no IgG immune response (figure 2).

### Cellular lymphoproliferation and cytokine response to PPD Rt 48

Cytokines were analysed for all subjects except subject 6 with the objective of observing Th_1 _(represented by IFN-γ) and Th2 (represented by IL-4) responses.

**Figure 3:**
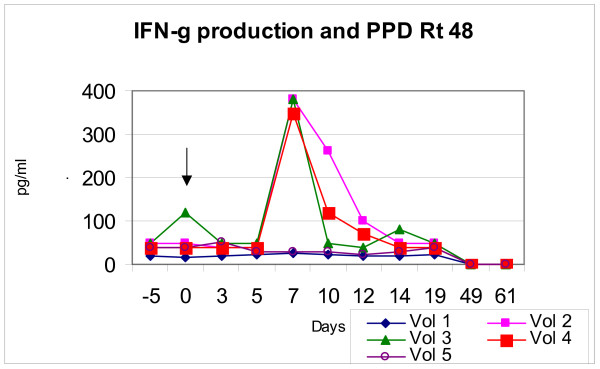
**IFN-γ production and PPD Rt 48**. Subjects displayed peak production of IFN-γ in response to PPD Rt 48 between days 5 and 12. Arrows indicate time of immunisation.

**Figure 4:**
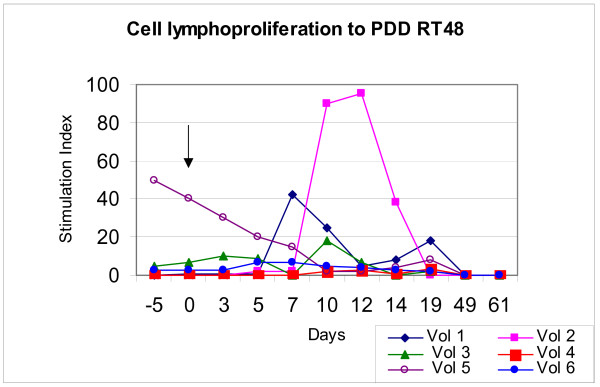
**Cell lymphoproliferation to PPD Rt 48**. All subjects showed a lymphoproliferative response to PPD Rt48 although the magnitude of stimulation differed. Arrows indicate time of immunisation.

All subjects showed a lymphoproliferative response to PPD Rt48 although the magnitude of stimulation differed (figures 3 and 4). Volunteers 1 to 5 showed production of IFN-γ and 1 to 6 showed lymphoproliferative responses to PPD Rt48. The peak production of IFN-γ was 30 pg/ml for volunteer 1, 330 pg/ml for volunteer 2, 380 pg/ml for volunteer 3, 40 pg/ml for volunteer 4 and 50 pg/ml for volunteer 5. The stimulation indices (SI = cpm tested: cpm control – RPMI medium) of the same subjects were respectively: 45, 90, 20, 4, 50 and 8.

With regard to cytokine production in response to secreted proteins, production of IFN-γ was observed, peaking at day 7 after oral immunisation. Levels of expression differed between individuals, but kinetics were maintained. All subjects were capable of IL-4 production in response to secreted proteins although no peak was observed; instead constant production of this cytokine was observed over the study period (data not shown).

## Discussion

Use of intradermal BCG vaccination results in a greater number of individuals capable of responding to skin testing, but the durability of this response varies between individuals and the diameter of induration diminishes with time. In some programmes, negative skin test response to PPD has been interpreted as an indication for revaccination. According to Hoft et al (2000) [20], immunisation with oral BCG inhibits DTH responses, but this inhibition does not represent a state of tolerance, since these individuals show significant mycobacteria-specific IFN-γ responses. Another hypothesis to explain the paucity or lack of DTH response in orally immunised individuals is that different populations of T cells are activated by BCG vaccination via the oral and intradermal routes [20].

We also observed that two individuals who received boosting with oral BCG vaccine during the study showed an alteration in humoral immune response seen as a shift in isotype from IgG to IgA, suggesting that oral revaccination is capable of provoking cellular and humoral responses. This response is independent of the route used in previous vaccination. Given that tuberculosis affects an important mucosal site, the respiratory tract, the potential use of oral booster vaccination in immunisation programmes is of interest. Subjects who were not boosted were not capable of mounting this shift in immunoglobulin isotype for the antigens tested. Hoft et al (2000) [20] propose a combination of oral and intradermal routes for BCG vaccination with the objective of inducing protective mucosal and systemic immunity against initial infection and systemic progression.

The kinetics of lymphoproliferative response described in this study are similar to those previously described using oral cholera vaccine [10]. Like our previous study, we can demonstrate that proliferation and trafficking of primed T-cells shows stimulation of the mucosal immune response at the same time as the systemic immune response; peak responses in this study (d7-12) are similar to those seen by Castello-Branco et al (1994) [10].

There were no differences in kinetics of T-cell circulation between PPD skin test responders and non-responders. All subjects developed a lymphoproliferative response after immunisation, suggesting the existence of circulating T-cells homing to the site where they were primed. Immunisation with *M. bovis BCG Moreau RDJ *was capable of altering the immune response to the cellular arm (Th_1_), critical for protection against infection, without failing to stimulate the humoral immune response (Th_2_) necessary for control of the cellular response [24,1].

It is important to remember that half the subjects reported contact with tuberculosis patients, and that contact with atypical mycobacteria could have influenced ELISpot results, an index of immune stimulation by mycobacterial antigens. Age, which varied between 18 and 50 years, could also have contributed to differences in responses.

A previous study (Das et al 1998) [14] of BCG vaccination in PPD skin test negative individuals showed a lack of IFN-γ response after immunisation. The strain of BCG used in immunisation may influence immune responses. As well as being is one of the most immunogenic BCG strains, *M. bovis BCG Moreau RDJ *shows great similarity to the original strain produced by Calmette and Guérin; only the BCG Russia strain genetically closer to the original, but is associated with a high incidence of adverse events including BCG osteitis [6]. These genetic strain differences are clearly of fundamental importance in determining immune responses as well as virulence [5].

This study demonstrates, for the first time, the immune response to oral immunisation with *M. bovis BCG Moreau RDJ *in humans. All subjects, including those who did not respond to the skin test at study commencement, were capable of mounting humoral and cellular immune responses to the antigens tested.

The data presented here will form the basis for further studies with larger numbers of subjects, with the aim of studying additional variables including immunoglobulins isotypes, other cytokines of importance in mucosal immune responses as well as responses to purified secreted proteins.

## Competing interests

The author(s) declare that they have no competing interests.

## Authors' contributions

RMM carried out the ELISpot, cellular proliferation and cytokine measurement assays, participated in the study design and wrote the manuscript. MBOS and LRCB recruited volunteers. MBOS and RTP participated in the study design and participated in the drafting of the manuscript. LRCB conceived the study, data analysis, coordination, the draft and finalisation of the manuscript. All authors read and approved the final manuscript.
